# Supplementary material for: Preparative Separation of Phenylethanoid and Secoiridoid Glycosides from Ligustri Lucidi Fructus by High-Speed Counter-Current Chromatography Coupled with Ultrahigh Pressure Extraction
Source: Molecules. 2018 Dec 18;23(12):3353. doi: 10.3390/molecules23123353 (PMC6321428; doi:10.3390/molecules23123353)
Supplement: Supplementary file 1 [file molecules-23-03353-s001.pdf]

Preparative Separation of Phenylethanoid and Secoiridoid Glycosides from Ligustri Lucidi Fructus by High-Speed Counter-Current Chromatography coupled with Ultrahigh Pressure Extraction

Fengwei He[1], Li Chen[1], Qian Liu[2], Xiao Wang[2], Jia Li[3,*] and Jinqian Yu [2,*]

[1] Liaoning Institute of Science and Technology, Benxi 117004, China; hfwdd@163.com (F. H.); 504373747@qq.com (L.C.)
[2] Shandong Key Laboratory of TCM Quality Control Technology, Shandong Analysis and Test Center, Qilu University of Technology (Shandong Academy of Sciences), Jinan 250014, China; 18765878227@126.com (Q.L.); wangx@sdas.org (X.W.); yujinqian87528@126.com (J.Y.)
[3] College of Pharmacy, Shandong University of Traditional Chinese Medicine, Jinan 250355, China; ljytl7172@163.com (J.L.)

* Correspondence: yujinqian87528@126.com; ljytl7172@163.com; Tel.: +86-0531-8260-5319(J.Y.); +86-0531-8962-8081 (J.L.);

Supplementary material

Table of Contents

**no.**	Content	Page
**1**	**Figure S1.** The HREIMS Spectroscopic Data of Compound **1**	S3
**2**	**Figure S2.** The ^{1}H NMR Spectrum of Compound **1** in DMSO-d_6 (400 MHz)	S4
**3**	**Figure S3.** The ^{13}C NMR Spectrum of Compound **1** in DMSO-d_6 (100 MHz)	S5
**4**	**Figure S4.** The HREIMS Spectroscopic Data of Compound **2**	S6
**5**	**Figure S5.** The ^{1}H NMR Spectrum of Compound **2** in Methanol-d_4 (400 MHz)	S7
**6**	**Figure S6.** The ^{13}C NMR Spectrum of Compound **2** in Methanol-d_4 (100 MHz)	S8
**7**	**Figure S7.** The HREIMS Spectroscopic Data of Compound **3**	S9
**8**	**Figure S8.** The ^{1}H NMR Spectrum of Compound **3** in Methanol-d_4 (400 MHz)	S10
**9**	**Figure S9.** The ^{13}C NMR Spectrum of Compound **3** in Methanol-d_4 (100 MHz)	S11
**10**	**Figure S10.** The HREIMS Spectroscopic Data of Compound **4**	S12
**11**	**Figure S11.** The ^{1}H NMR Spectrum of Compound **4** in Methanol-d_4 (400 MHz)	S13
**12**	**Figure S12.** The ^{13}C NMR Spectrum of Compound **4** in Methanol-d_4 (100 MHz)	S14
**13**	**Figure S13.** The HREIMS Spectroscopic Data of Compound **5**	S15
**14**	**Figure S14.** The ^{1}H NMR Spectrum of Compound **5** in Methanol-d_4 (400 MHz)	S16
**15**	**Figure S15.** The ^{13}C NMR Spectrum of Compound **5** in Methanol-d_4 (100 MHz)	S17
**16**	**Figure S16.** The HREIMS Spectroscopic Data of Compound **6**	S18
**17**	**Figure S17.** The ^{1}H NMR Spectrum of Compound **6** in Methanol-d_4 (400 MHz)	S19
**18**	**Figure S18.** The ^{13}C NMR Spectrum of Compound **6** in Methanol-d_4 (100 MHz)	S20

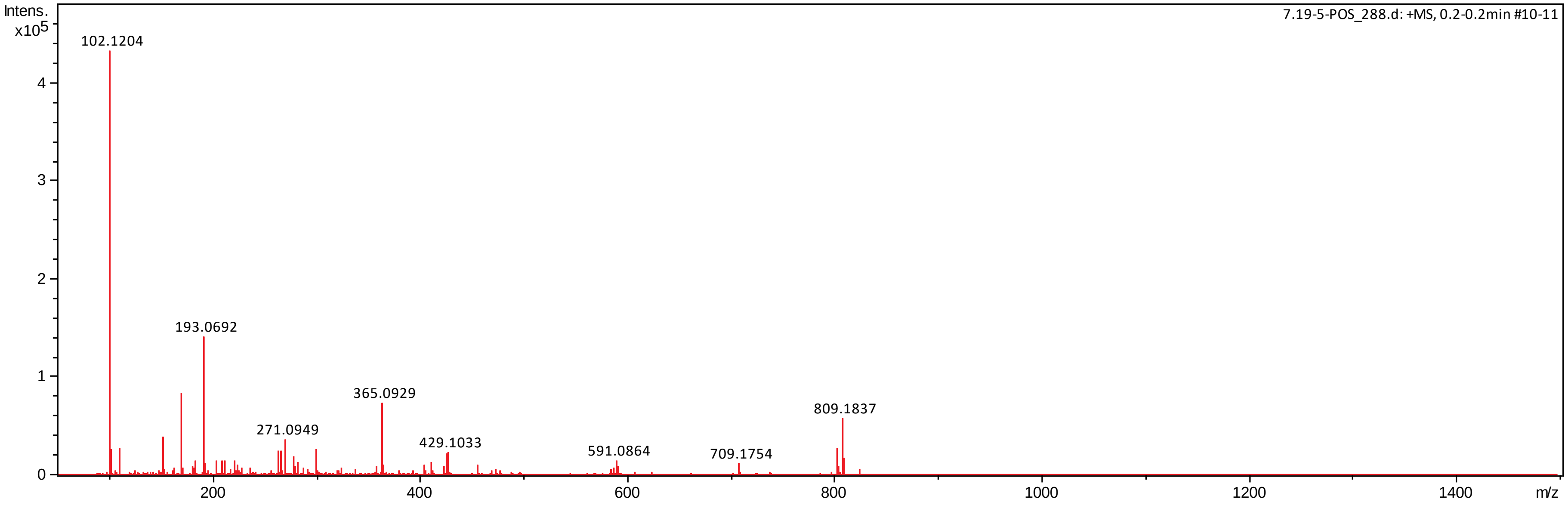

Figure S1. The HREIMS Spectroscopic Data of Compound **1**

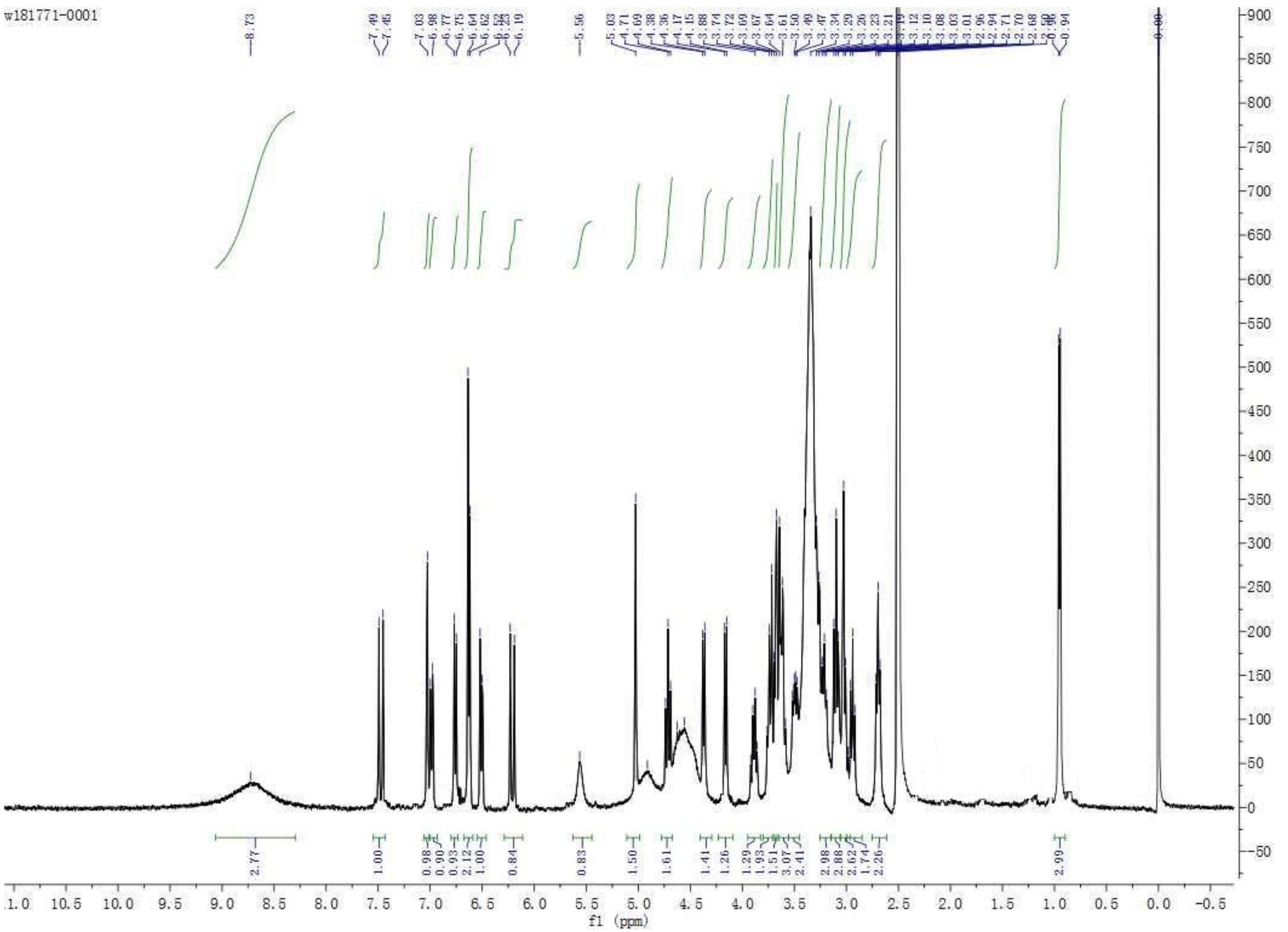

Figure S2. The ^{1}H NMR Spectrum of Compound **1** in DMSO-d_6 (400 MHz)

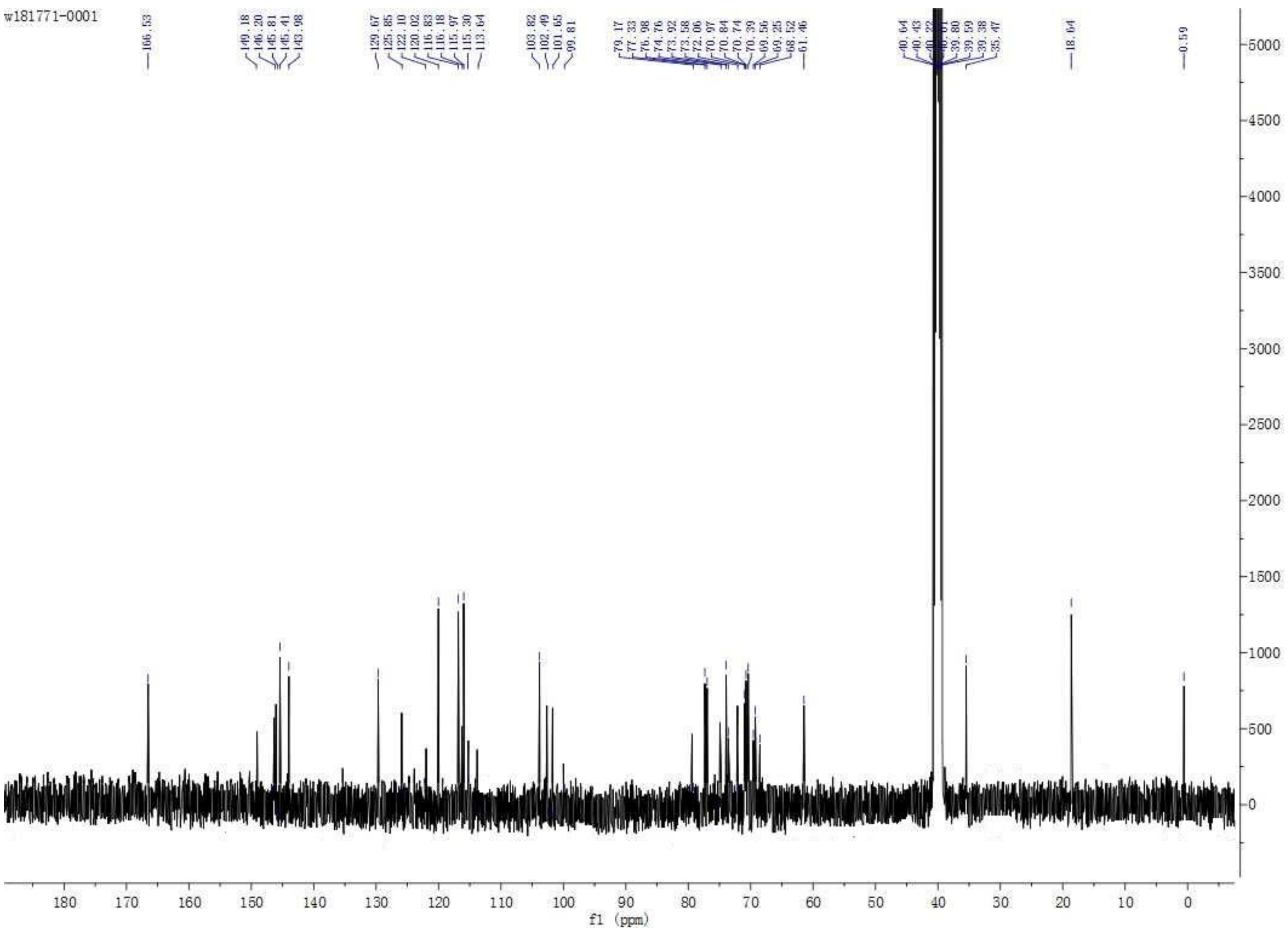

Figure S3. The ^{13}C NMR Spectrum of Compound **1** in DMSO-d_6 (100 MHz)

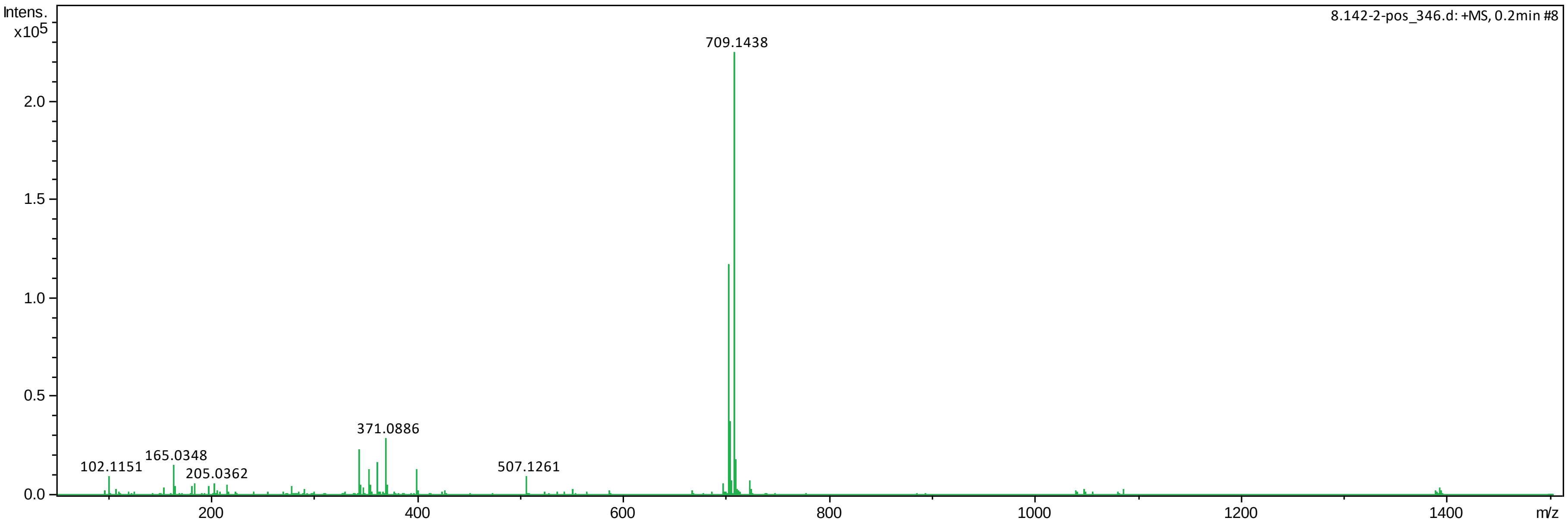

Figure S4. The HREIMS Spectroscopic Data of Compound **2**

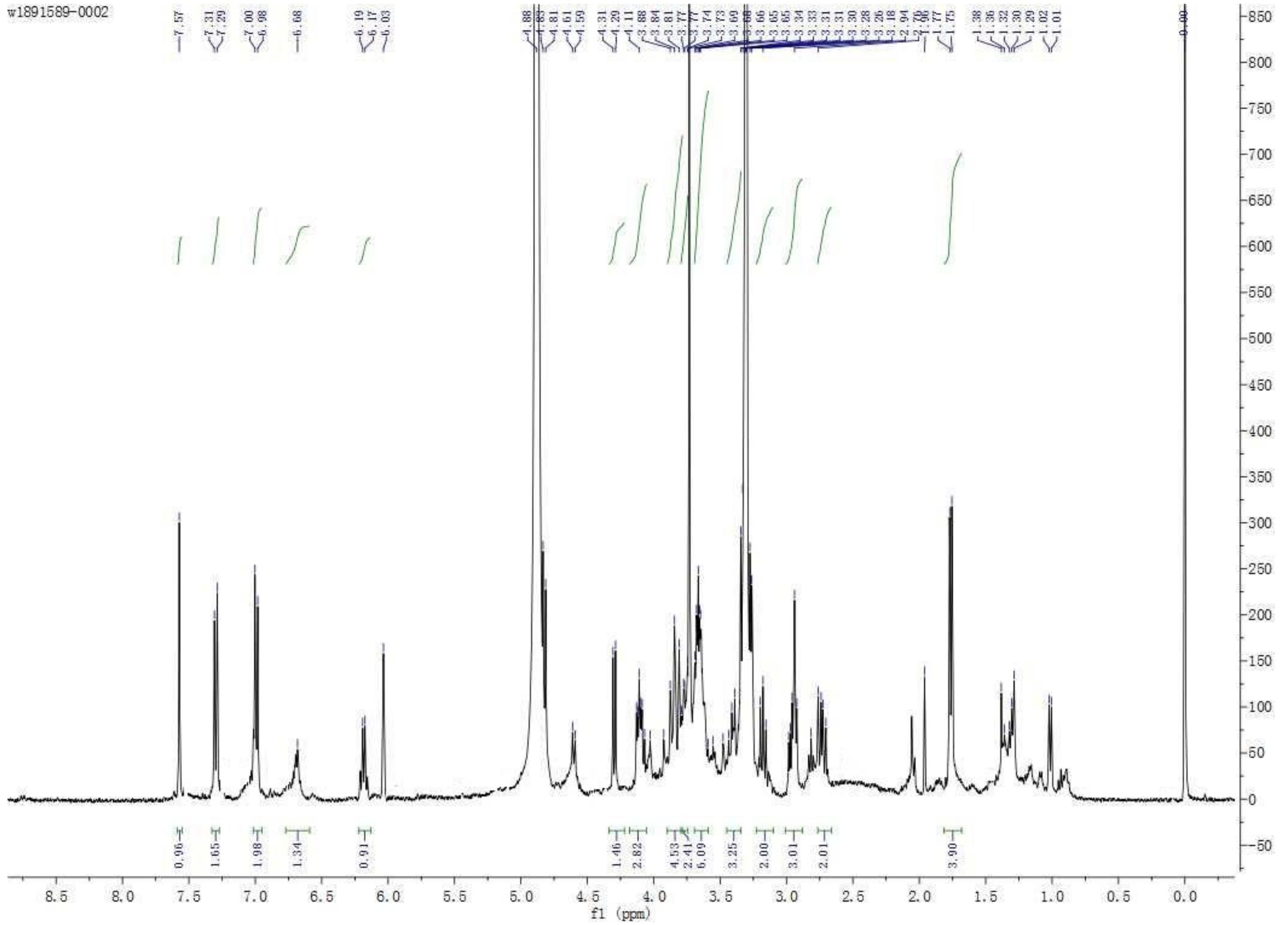

Figure S5. The ^{1}H NMR Spectrum of Compound **2** in Methanol-d_4 (400 MHz)

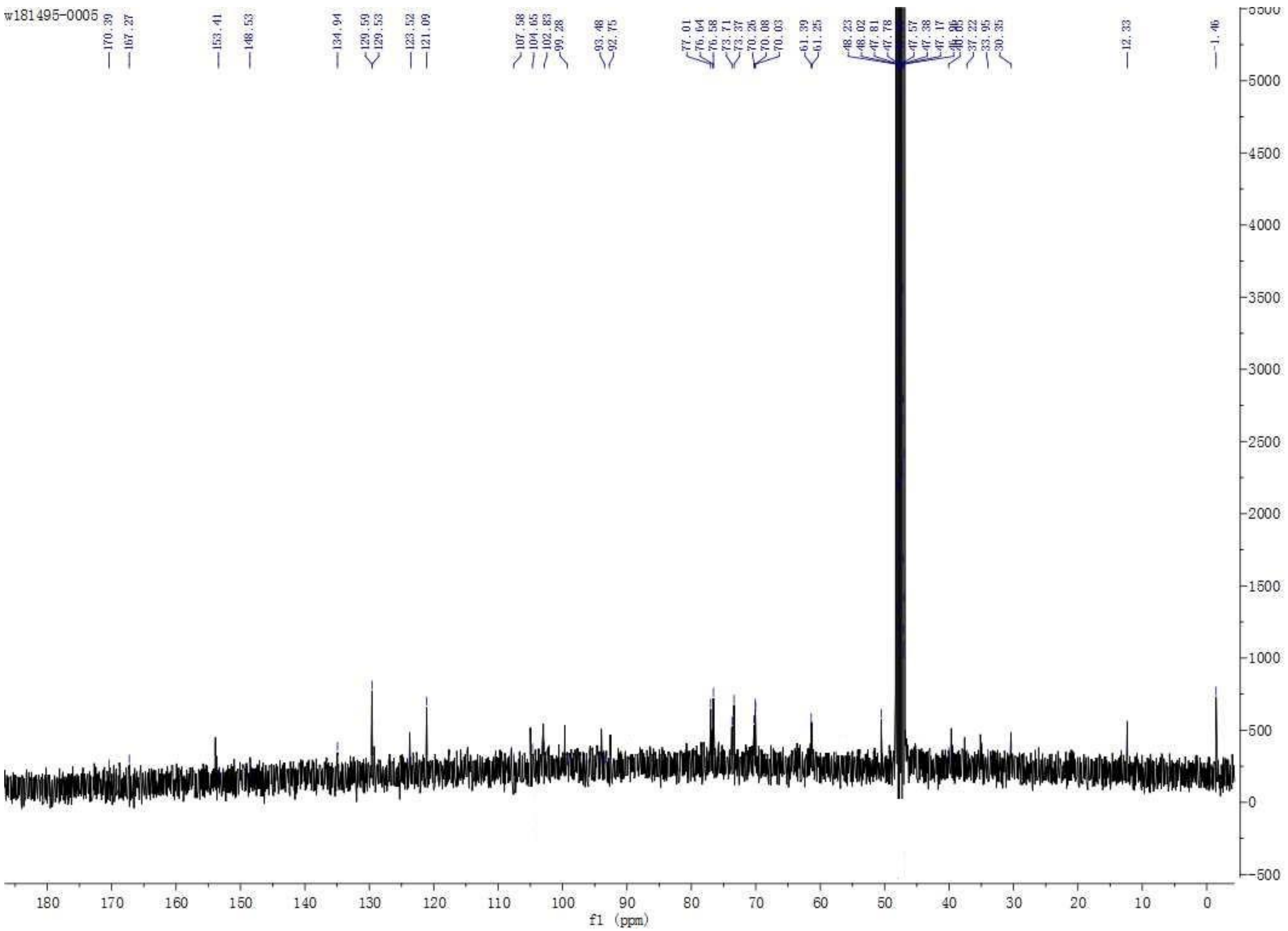

Figure S6. The ^{13}C NMR Spectrum of Compound **2** in Methanol-d_4 (100 MHz)

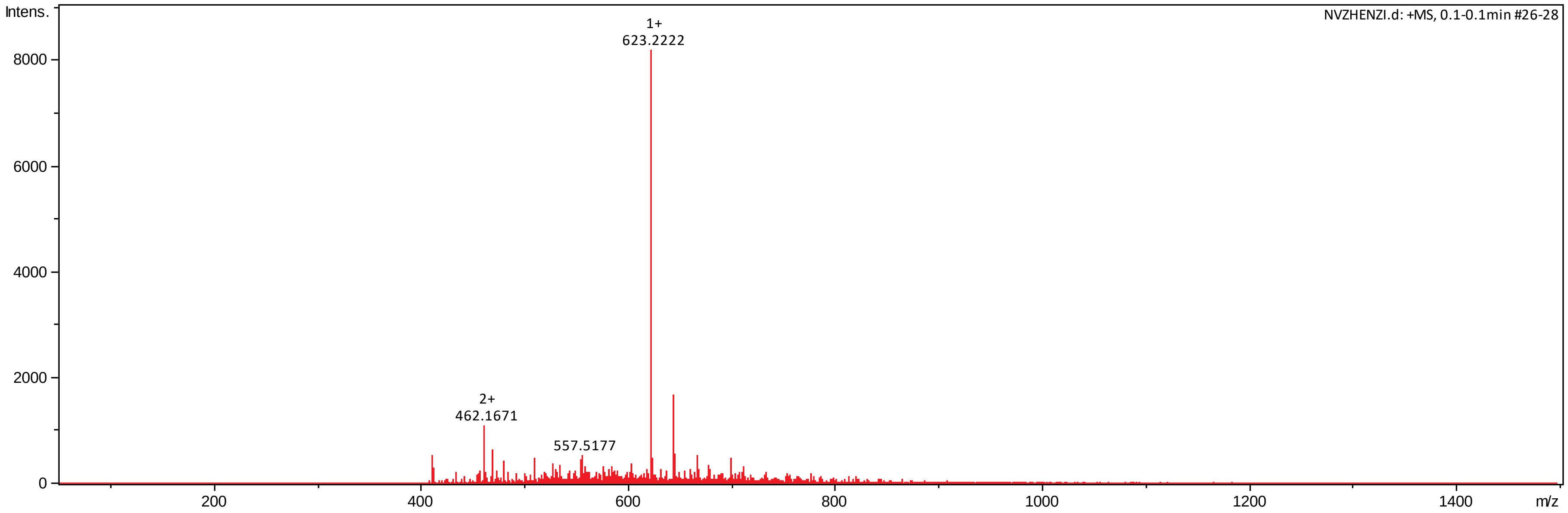

Figure S7. The HREIMS Spectroscopic Data of Compound **3**

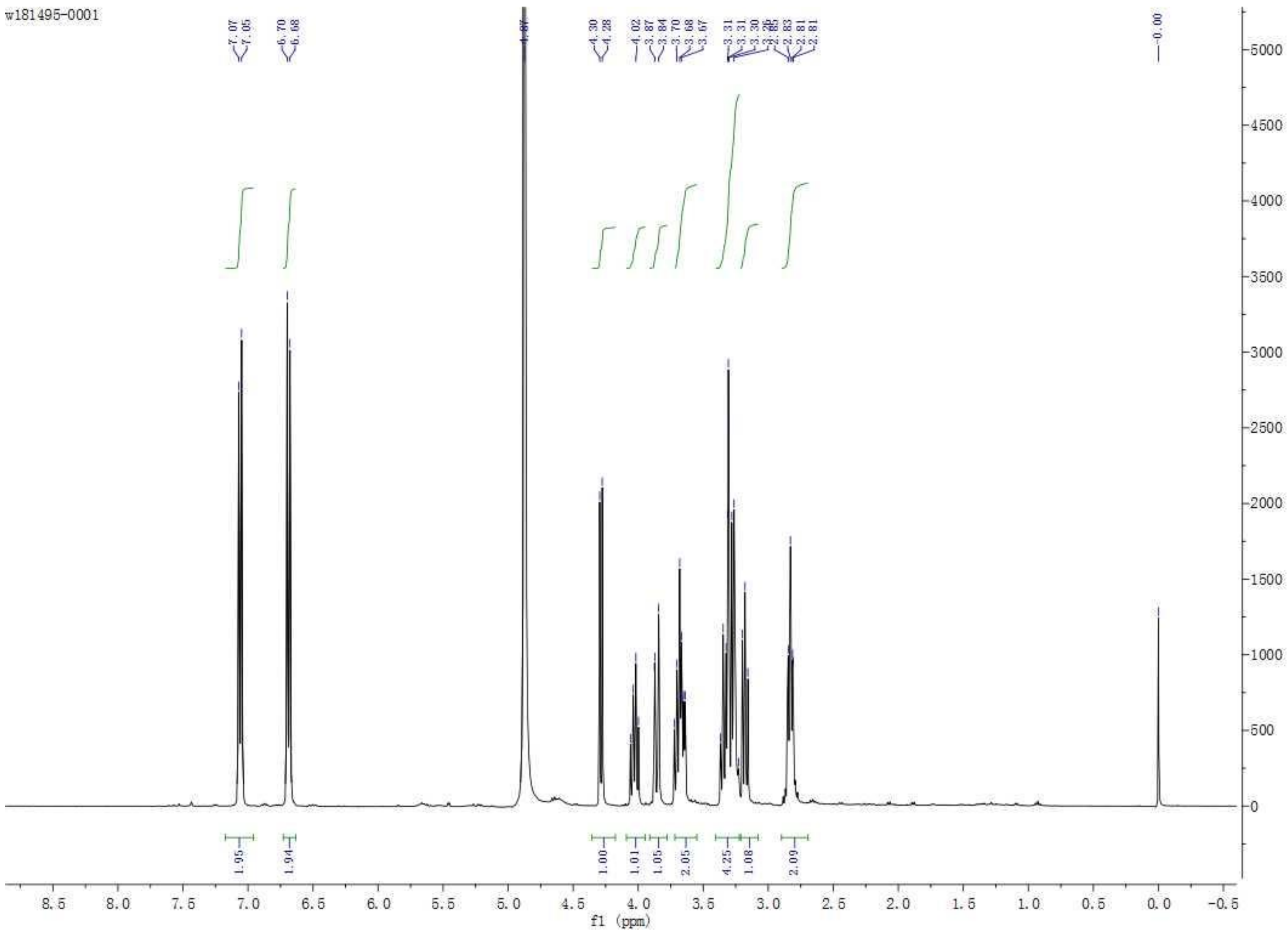

Figure S8. The ^{1}H NMR Spectrum of Compound **3** in Methanol-d_4 (400 MHz)

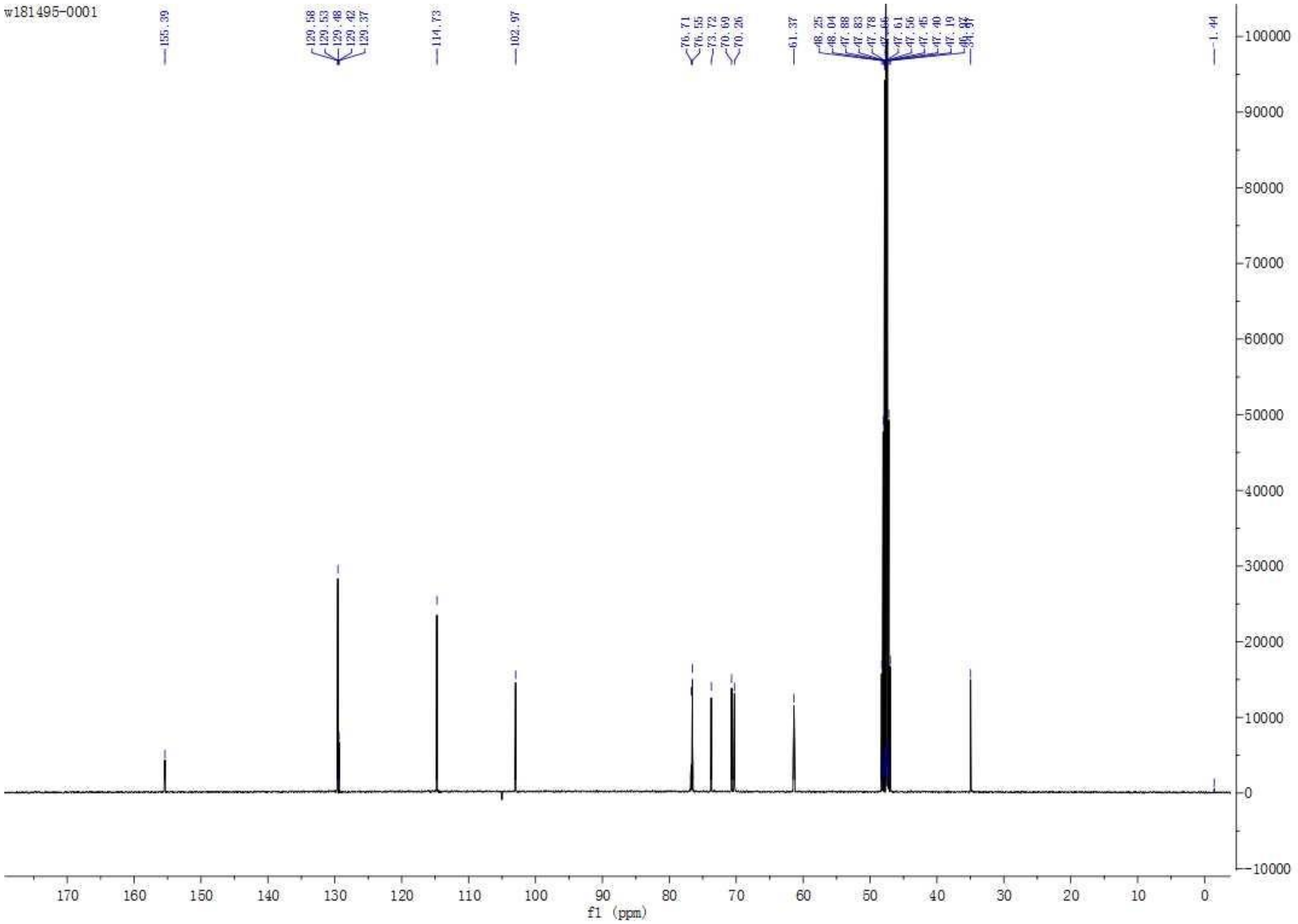

Figure S9. The ^{13}C NMR Spectrum of Compound **3** in Methanol-d_4 (100 MHz)

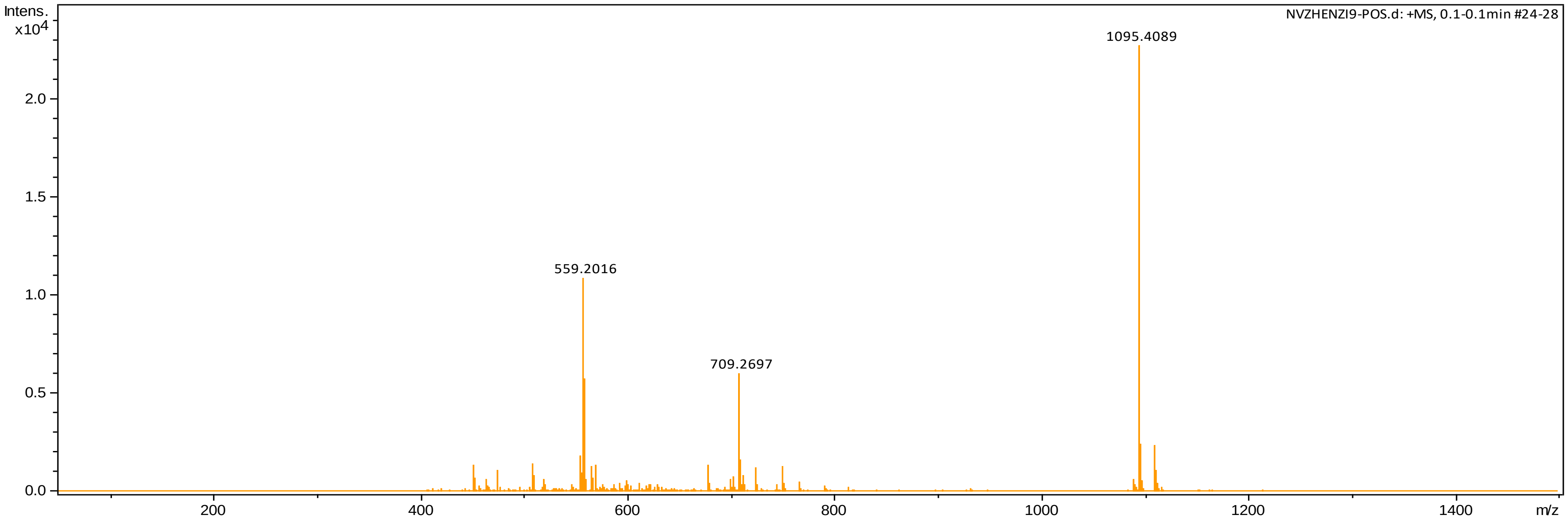

Figure S10. The HREIMS Spectroscopic Data of Compound **4**

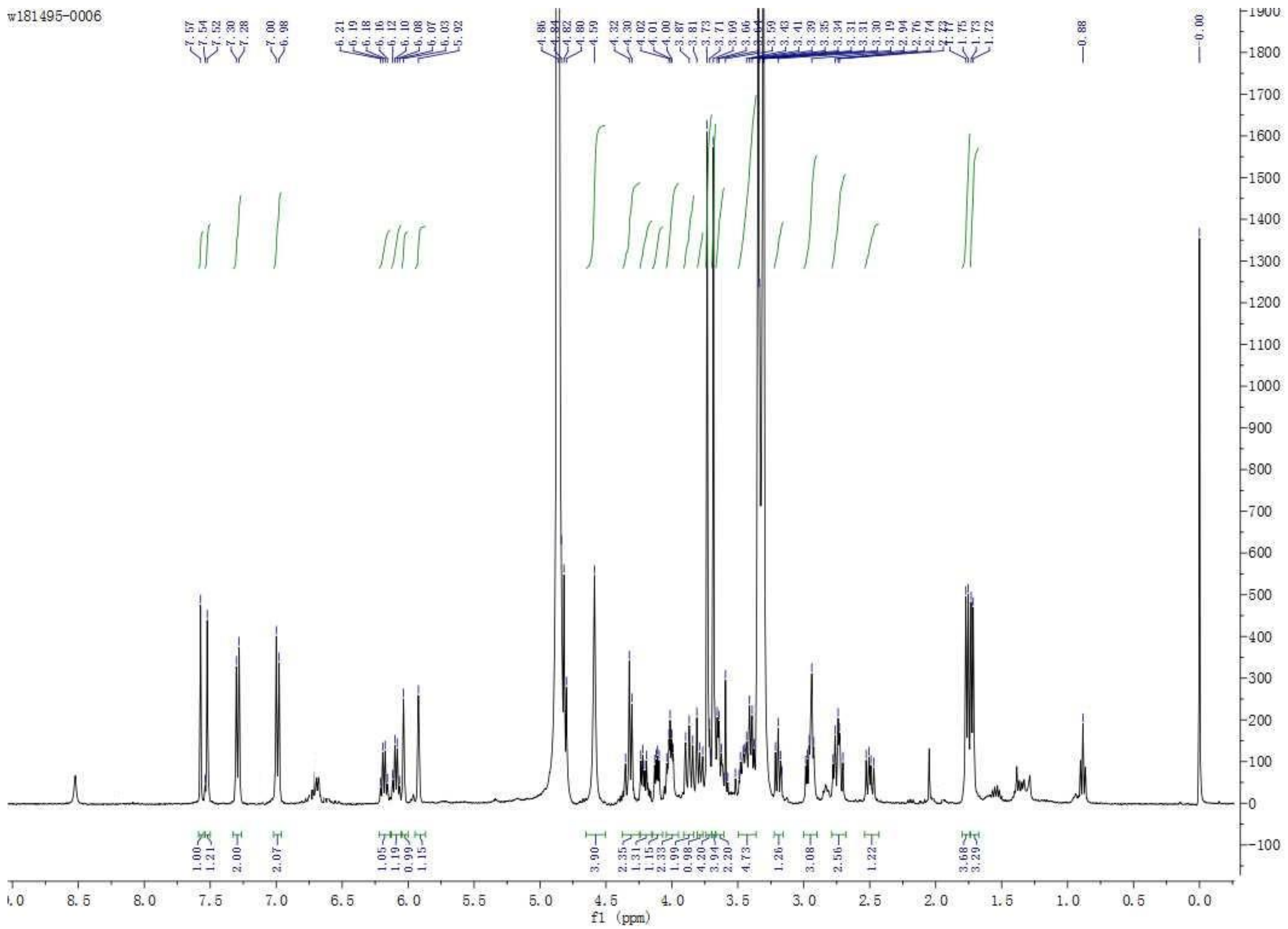

Figure S11. The ^{1}H NMR Spectrum of Compound **4** in Methanol-d_4 (400 MHz)

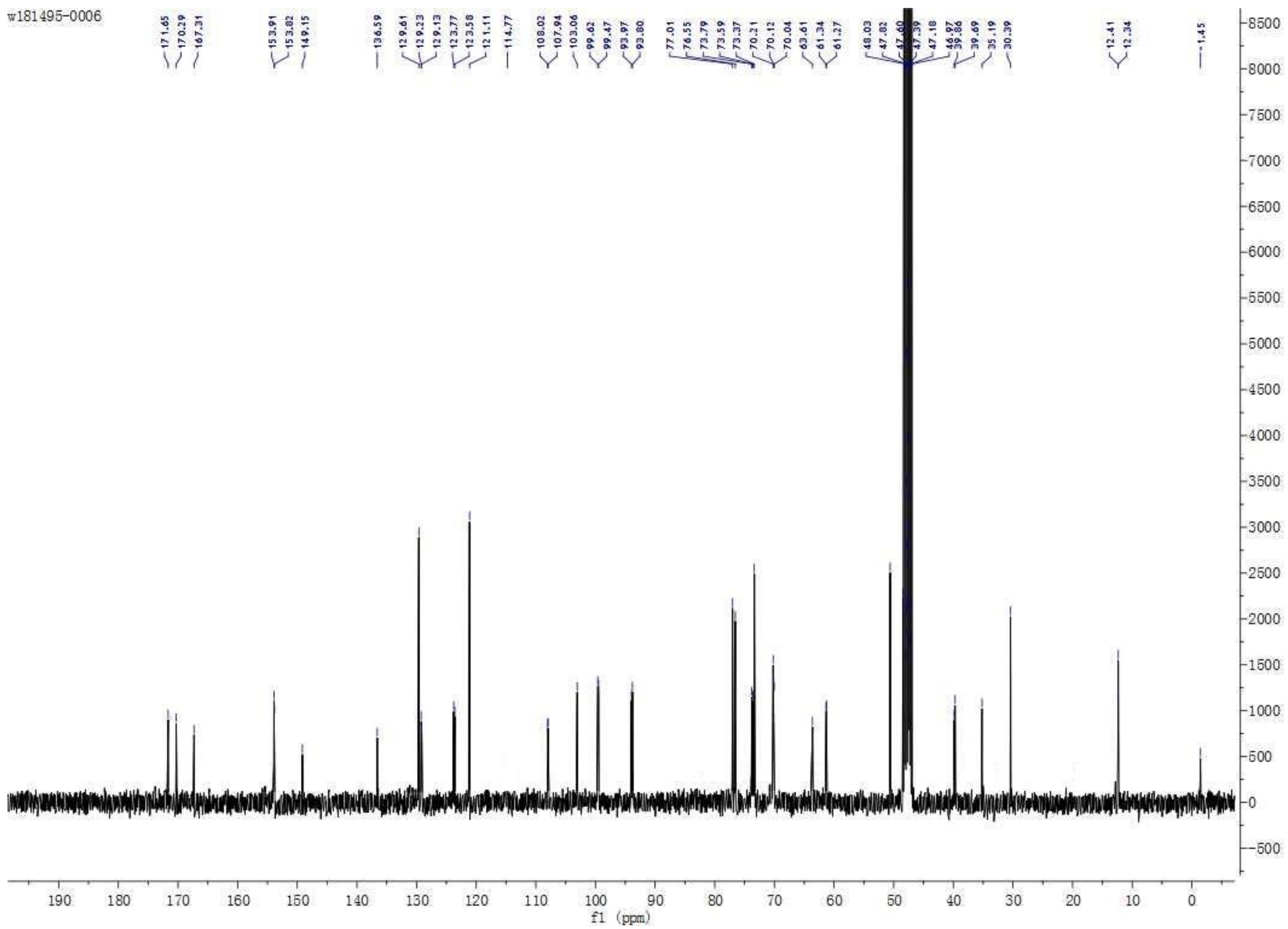

Figure S12. The ^{13}C NMR Spectrum of Compound **4** in Methanol-d_4 (100 MHz)

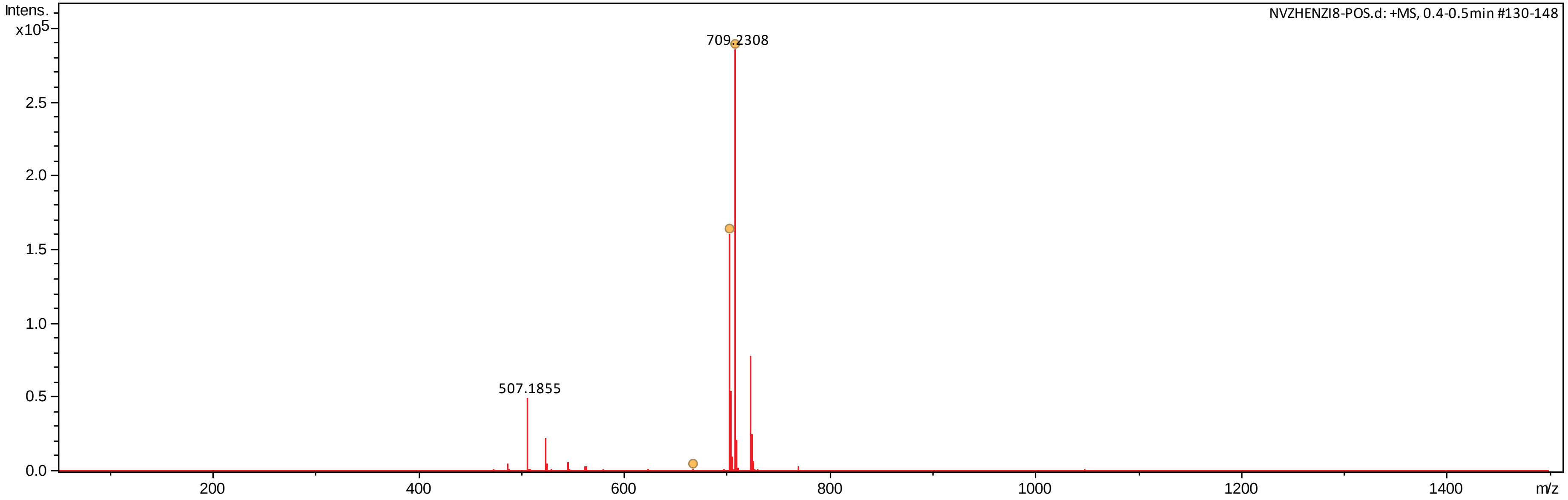

Figure S13. The HREIMS Spectroscopic Data of Compound **5**

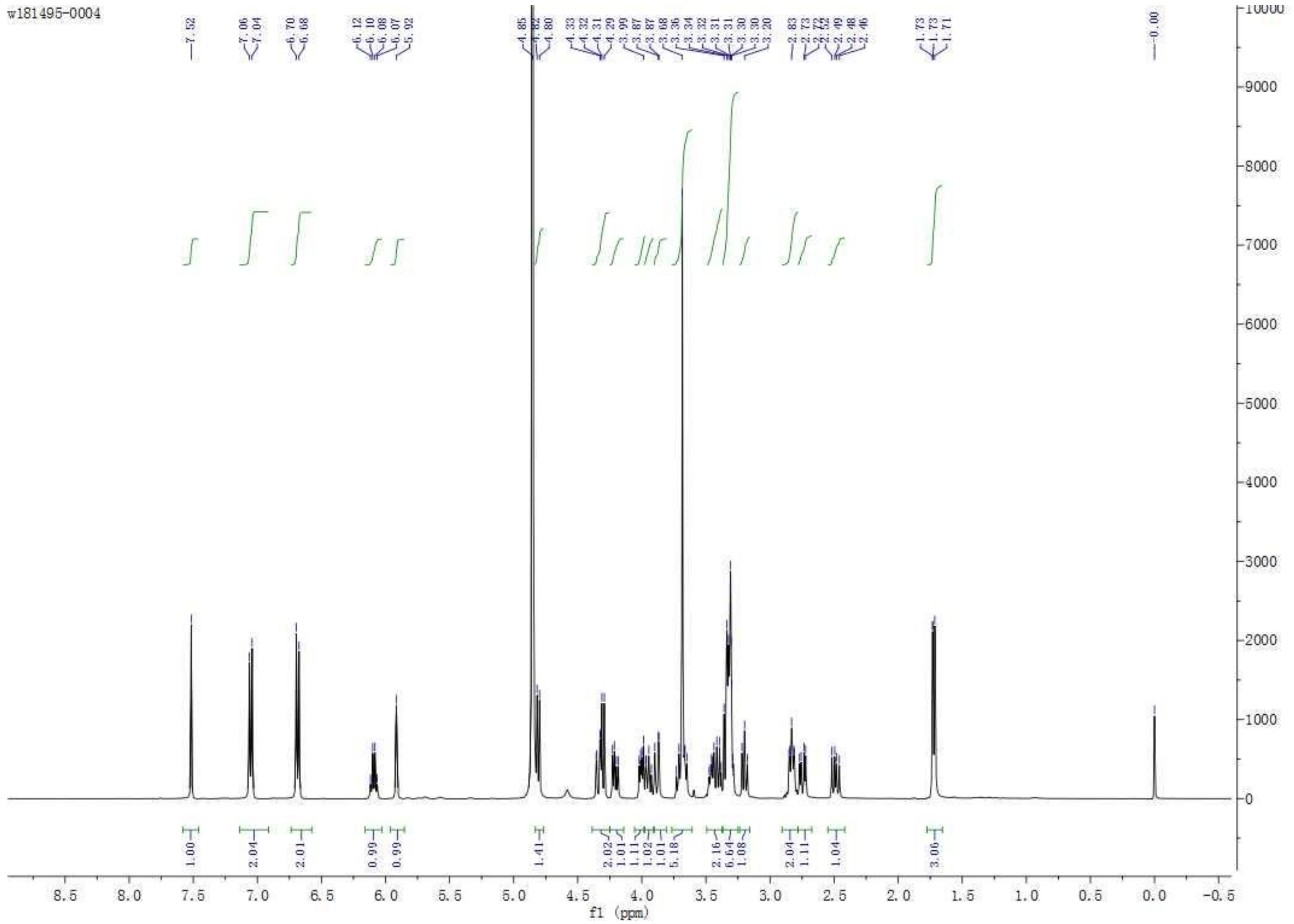

Figure S14. The ^{1}H NMR Spectrum of Compound **5** in Methanol-d_4 (400 MHz)

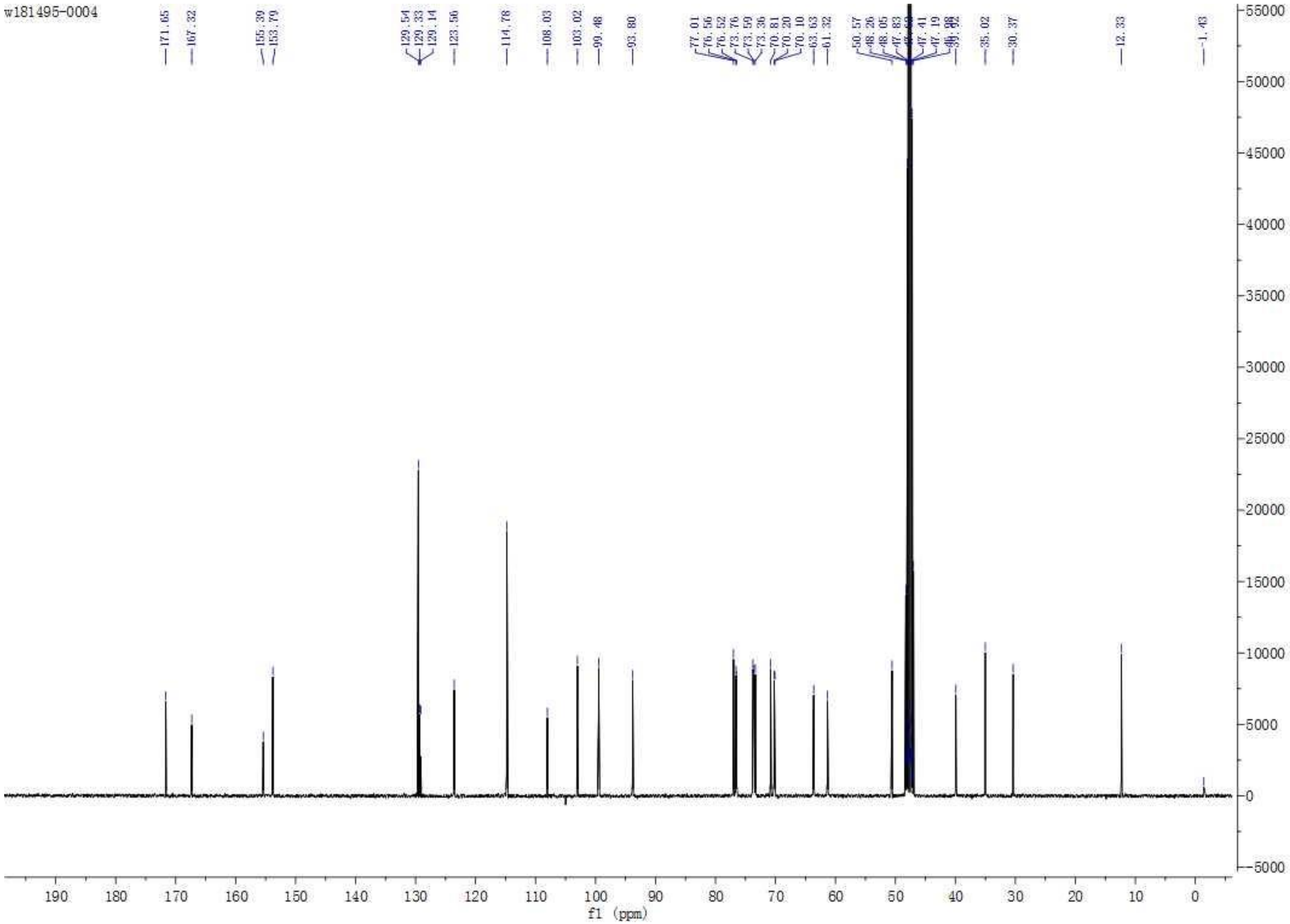

Figure S15. The ^{13}C NMR Spectrum of Compound **5** in Methanol-d_4 (100 MHz)

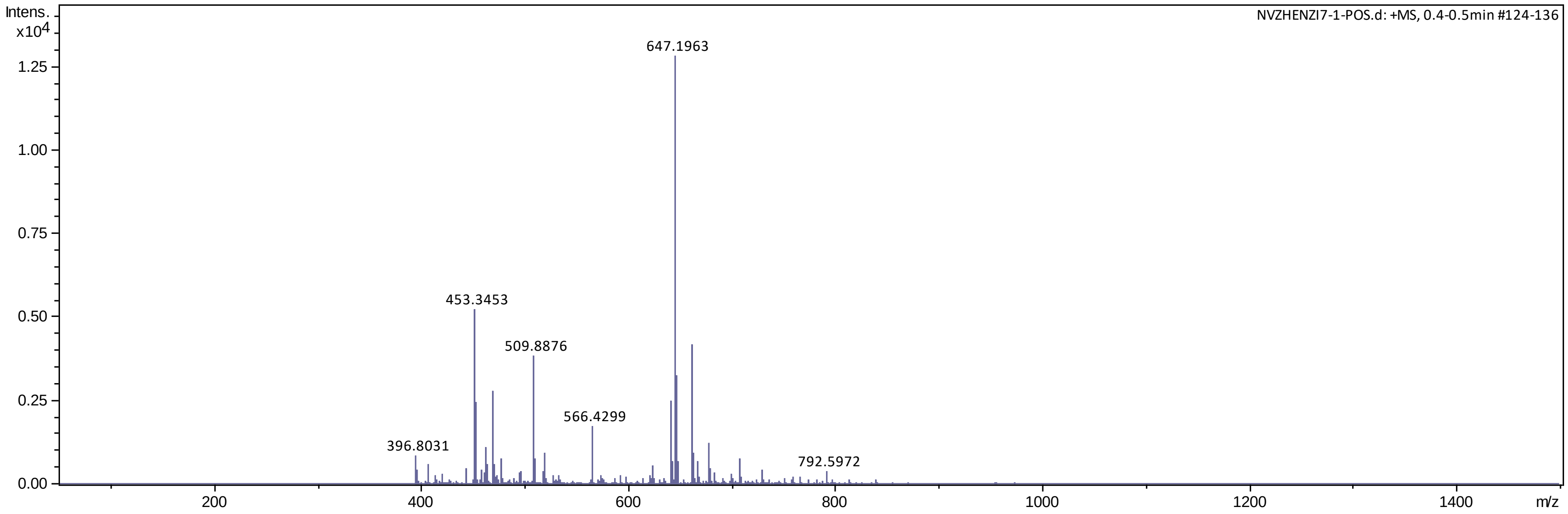

Figure S16. The HREIMS Spectroscopic Data of Compound **6**

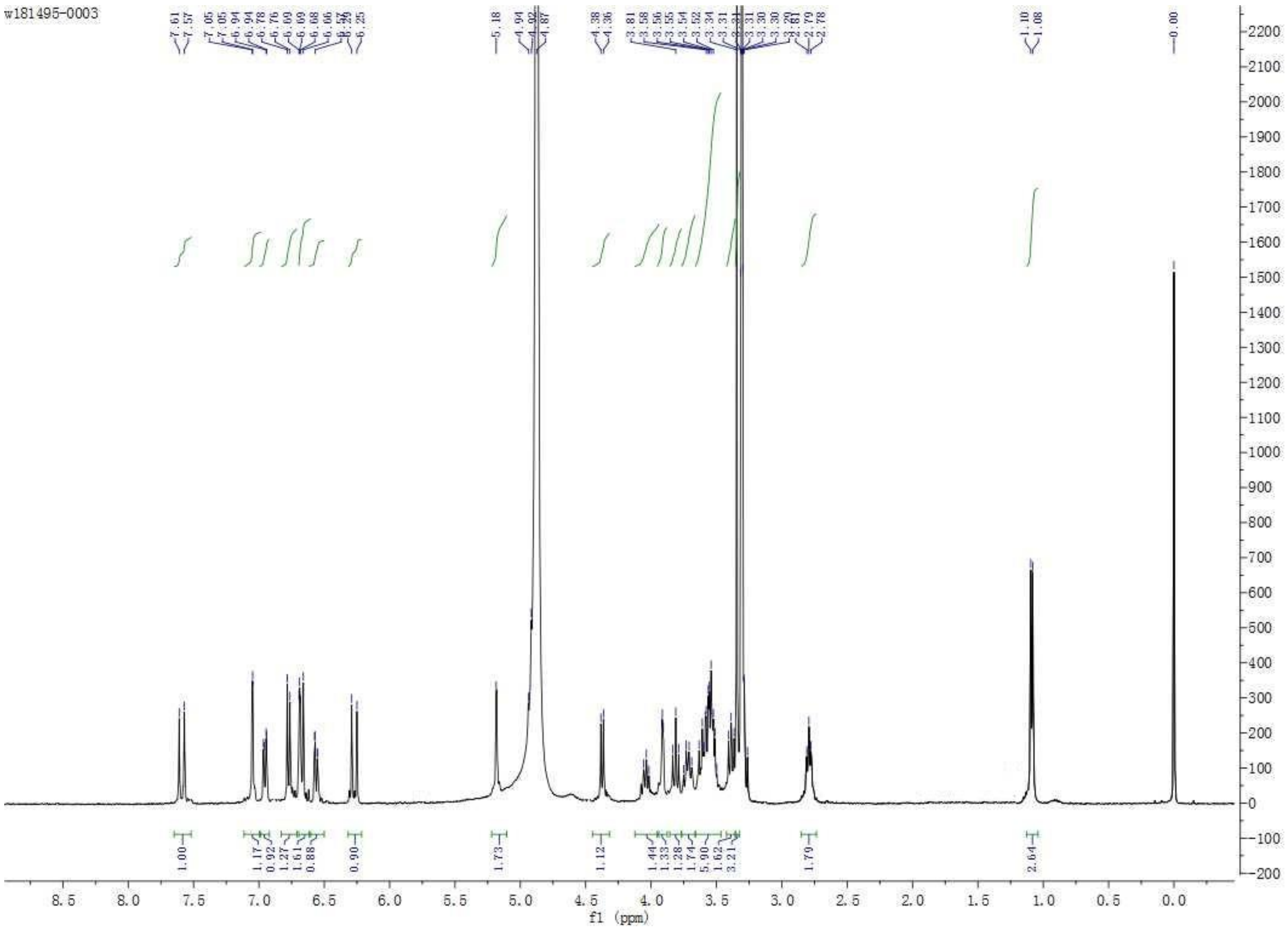

Figure S17. The ^{1}H NMR Spectrum of Compound **6** in Methanol-d_4 (400 MHz)

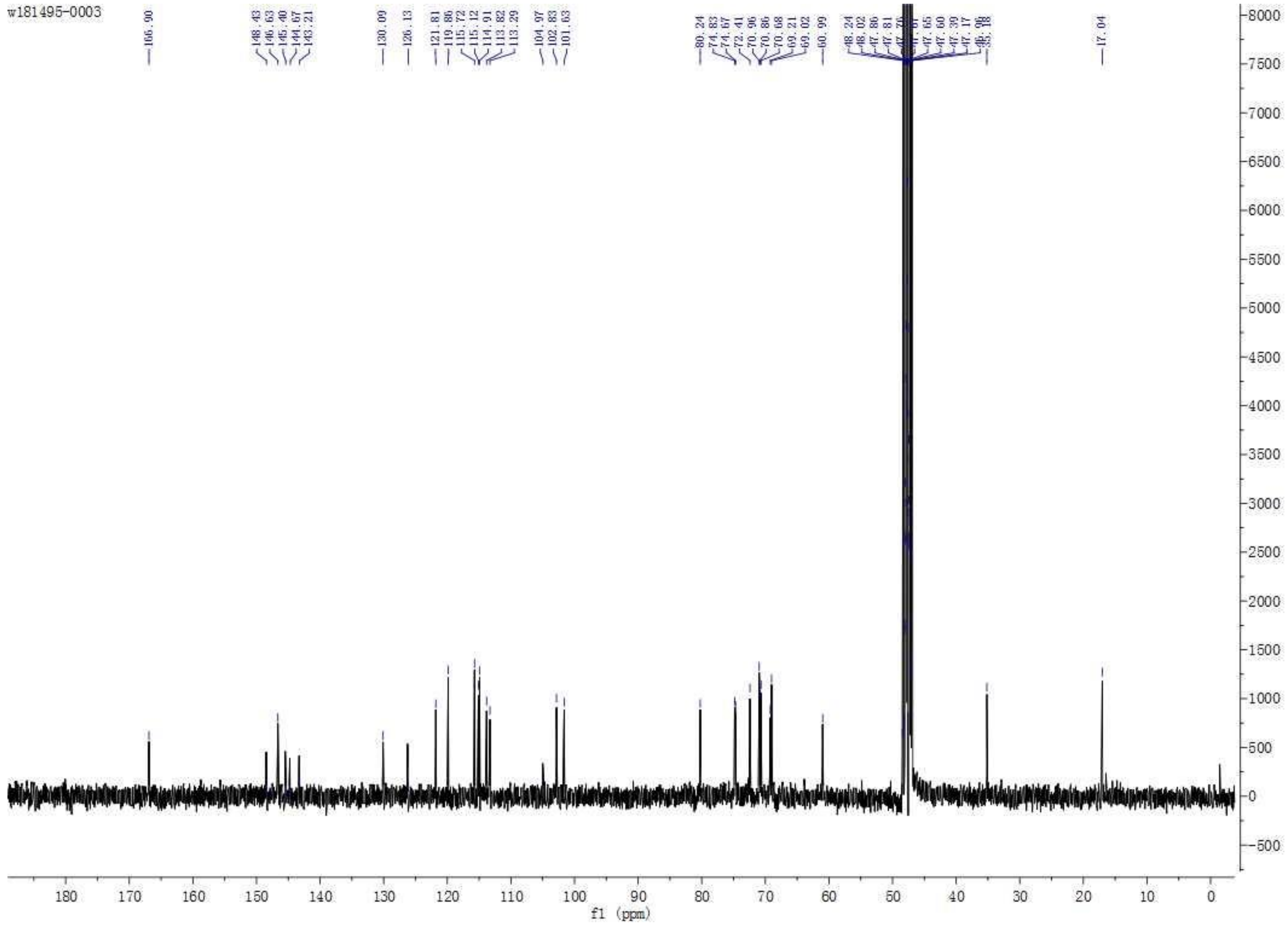

Figure S18. The ^{13}C NMR Spectrum of Compound **6** in Methanol-d_4 (100 MHz)
